# Prognostic value of systemic immune-inflammation index and serological biomarkers for deep neck infections

**DOI:** 10.4317/medoral.26130

**Published:** 2023-10-12

**Authors:** Jose Luis Treviño-Gonzalez, Felipe Acuña-Valdez, Karla M Santos-Santillana

**Affiliations:** 1 Otolaryngology and Head and Neck Surgery Division, School of Medicine and University Hospital “Dr. Jose E. González”, Universidad Autónoma de Nuevo León, Monterrey, Nuevo Leon, Mexico

## Abstract

**Background:**

Inflammatory biomarkers, including C-reactive protein, erythrocyte sedimentation rate, neutrophil to lymphocyte ratio, platelet to lymphocyte ratio, and the systemic immune-inflammation index, have been proposed as prognostic factors diverse pathologies. However, their application for deep neck infections has yet to be clarified.

**Material and Methods:**

We performed a retrospective study of 163 adult patients with diagnosis of deep neck infections with the aim to evaluate the association between serological biomarkers with complications and outcomes of patients with DNI. Studied variables included demographic data, complications of DNI, outcomes, complications and death of the included subjects. The evaluated serological biomarkers were hemoglobin, leukocytes, neutrophils, lymphocytes, platelets, glucose, creatinine, albumin, CRP, and ESR. NLR, PLR, and SIII index were estimated.

**Results:**

The patients’ mean age was 40.6 ± 15.3 years. Complications of DNI were observed in 19.6% (*n*=32) patients, being the need for tracheostomy due to airway obstruction (11%, *n*=18) and mediastinitis (8.6%, *n*= 14) the most common. Evaluated subjects had an increased value of serological biomarkers (SII index 2639.9 ± 2062.9, NLR 11.3 ± 8.5, PLR 184.1 ± 108.5, CRP 12.6 ± 8.9 mg/dL, ESR 20.7 ± 9.1 mm/h). Patients with complications had a significantly higher value of all inflammatory parameters (*p* < 0.05). A SII index cut-off value of 2975 was selected from a ROC curve analysis. A sensitivity of 93.8%, specificity of 86.3%, a positive predictive value of 62.5%, and a negative predictive value of 98.3% are reported. The SII index was found to have an increased positive predictive value compared to NLR, PLR, and CRP for DNI complications.

**Conclusions:**

Our analysis concluded that the SII index, NLR, and PLR are valuable biomarkers to assess the risk of complications from DNI. SII index showed a high accuracy for prediction of DNI complications with a cut-off value of 2975.

** Key words:**Neck infections, neck abscess, biomarkers, neutrophils, lymphocytes, systemic inflammation.

## Introduction

Deep neck infections (DNI) are a common pathology characterized by a prompt infection progression involving deep cervical spaces or fascial planes of the neck (1). DNI are associated with morbid and lethal complications, such as mediastinitis, septic thrombophlebitis, pericarditis, and obstructed airway, among others (2). The odontogenic origin of DNI remains the most common cause, reported between 20.5% to 88.7% (3-5). Other causes include acute pharyngeal infections, cervicofacial trauma, a bacterial infection of branchial arch remnants, and tuberculosis (4).

Several clinical and serological factors have been studied to determine severity, prognosis, complications, need for surgical debridement, and death in patients with DNI. The presence of dyspnea, stridor, trismus, and crepitus have been described as signs of severity and the need for hospitalary admission (6). Inflammatory biomarkers of severity have been proposed as prognostic factors in sepsis and infections, including C-reactive protein (CRP) (7), erythrocyte sedimentation rate (ESR) (6), neutrophil to lymphocyte ratio (NLR) (8,9), mean platelet volume (MPV) (10), platelet to lymphocyte ratio (PLR) (11), and the systemic immune-inflammation index (SII index) (12). However, their application for DNI has yet to be clarified.

The aim of this study is to evaluate the association between serological biomarkers, including hemoglobin, leukocytes, neutrophils, lymphocytes, platelets, glucose, creatinine, albumin, CRP, ESR, NLR, PLR, and SII index with complications and outcomes of patients with DNI.

## Material and Methods

This retrospective study was conducted in the University Hospital “Dr. Jose E. González”. The research protocol was approved by the local Research and Institutional Ethics Committee. The authors assert that all procedures contributing to this work comply with the ethical standards of the relevant national and institutional guidelines on human experimentation and with the Helsinki Declaration of 1975, as revised in 2008.

- Subjects

This study included patients over 18 years old with a diagnosis of DNI in the hospital database with the International Statistical Classification of Diseases 10th revision (ICD-10) code L02.1. Exclusion criteria included pregnancy, superficial neck infections, surgical or penetrating infected neck wounds, congenital neck malformations, patients with a history of neck surgery, head and neck cancer, radiotherapy, chemotherapy, incomplete medical records, and loss of follow-up after discharge. Data were extracted from medical records using a standardized data collection form. All authors contributed to data retrieval, and an independent author adjudicated any difference in interpretation between the data extractors.

- Anthropometric and biochemical measures

Studied variables included demographic data, days of stay, number of involved neck spaces, complications of DNI, need for surgical reintervention, outcomes, and death of the included subjects. The evaluated serological biomarkers were hemoglobin, leukocytes, neutrophils, lymphocytes, platelets, glucose, creatinine, albumin, CRP, and ESR. NLR (13) and PLR (14) were estimated as previously described. SII index was calculated according to the formula proposed by Hu *et al*. (SII = NLR × platelets) (15).

Radiological evaluation was assessed by a head and neck radiologist by computed tomographies obtained from the University Hospital “Dr. Jose E. González” Diagnostic Radiology Department’s database.

- Statistical analysis

Statistical analysis was performed using SPSS V25.0 (Armonk, NY: IBM Corp). Categorical variables are reported as percentages and frequencies; continuous variables are reported as means and standard deviations. Categorical variables were compared using Pearson’s x2 test or Fisher’s exact test for 2 x 2 Tables. An unpaired Student’s t test or Mann-Whitney U test were used to compare continuous variables. *P* < 0.05 was considered statistically significant. ROC curves were performed to determine the sensitivity, specificity, positive and negative predictive value of the SII index, LNR, PLR and CRP. Spearman's coefficient correlation test was used to determine the correlation of SII index to distinct parameters of systemic inflammation.

## Results

A total of 163 patients with a diagnosis of DNI were included in the study. The patients mean age was 40.6 ± 15.3 years. The most common comorbidity was diabetes mellitus in 30.7% (*n*=50) of cases. The primary cause of DNI were odontogenic infections (82%, *n*= 139), pharyngeal infections (12.9%, *n*= 21), and neck and mandibular trauma (4.9%, *n*= 8). Bilateral involvement of DNI was seen in 7.4% (*n*= 12) of cases. Complications of DNI were observed in 19.6% (*n*=32) patients, being the need for tracheostomy due to airway obstruction (11%, *n*=18) and mediastinitis (8.6%, *n*= 14) the most common. The mortality rate was 5.5% (*n*=9) (Table 1).

- Inflammatory serological biomarkers

Clinical and serological characteristics were compared between patients with and without complications. Overall, evaluated subjects had an increased value of serological biomarkers (SII index 2639.9 ± 2062.9, NLR 11.3 ± 8.5, PLR 184.1 ± 108.5, CRP 12.6 ± 8.9 mg/dL, ESR 20.7 ± 9.1 mm/h). Patients with complications had a significantly higher value of leucocytes, neutrophils, SII index, NLR, PLR, CRP, and ESR (*p* < 0.05) (Table 2).

**Table 1:**
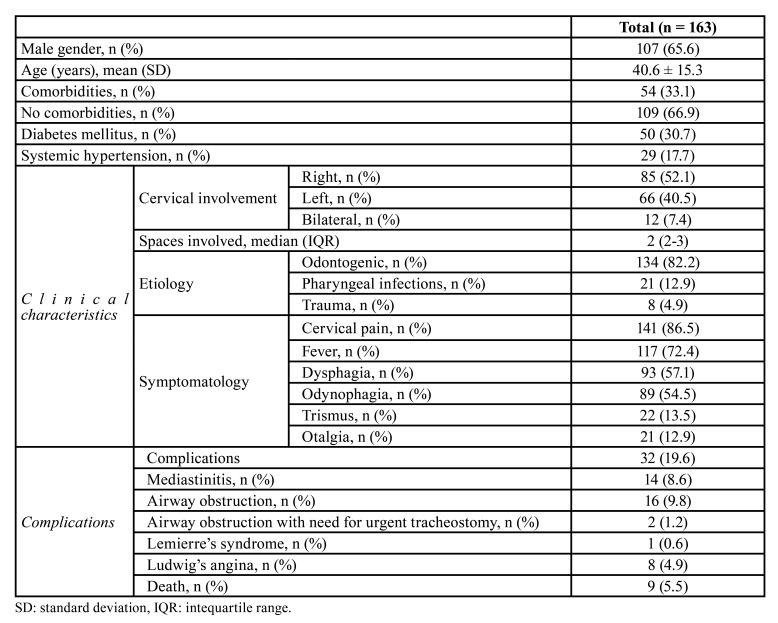
Patient demographics, clinical characteristics, and outcomes.

**Table 2:**
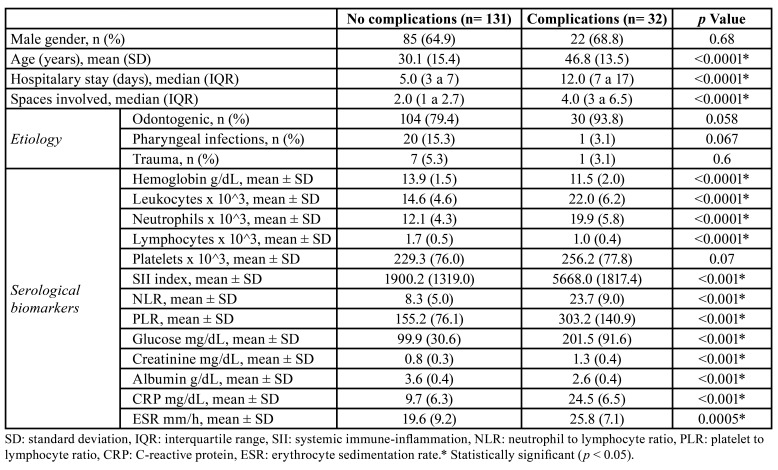
Clinical characteristics and serological data associated with deep neck infections complications.

- Systemic Immune-Inflammation Index as a predictor for complications

**Figure 1:**
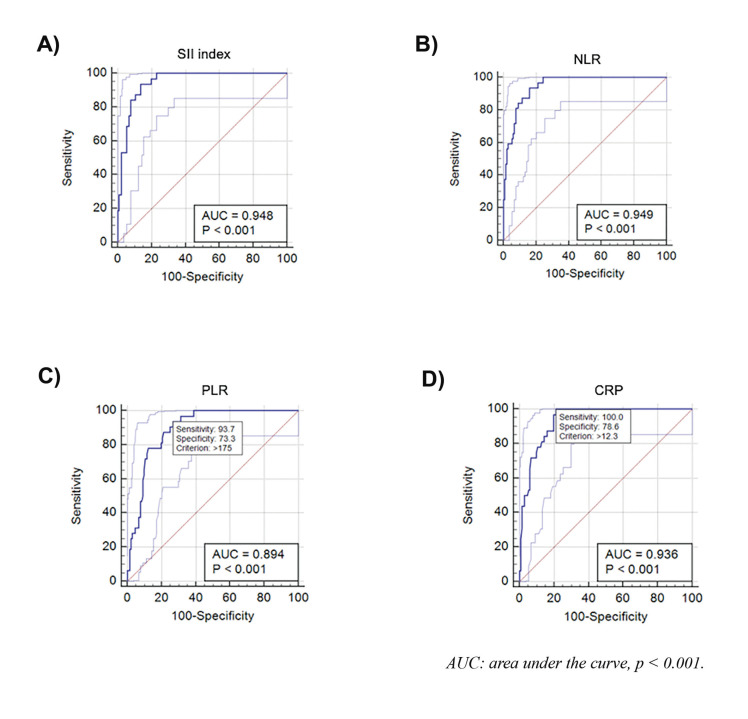
ROC curve, sensitivity represents true-positive results, specificity-1 represents the false-positive results. A) The best cut-off value of SII index for high-risk of DNI complications was 2975 in the evaluated patients. B) NLR cut-off value for high-risk of DNI complications was 11. C) PLR cut-off value for high-risk of DNI complications was 175. D) CRP cut-off value for high-risk of DNI complications was 12.3.

A SII index cut-off value of 2975 was selected from a ROC curve analysis. A sensitivity of 93.8%, specificity of 86.3%, a positive predictive value of 62.5%, and a negative predictive value of 98.3% are reported. The SII index was found to have an increased positive predictive value compared to NLR, PLR, and CRP for DNI complications (Fig. 1). Efficiency of serological biomarkers can be observed in Table 3. A higher value of the SII index was associated with an increased risk of mediastinitis, airway obstruction, need for surgical reintervention, and death (*p* <0.001) (Table 4).

**Table 3:**
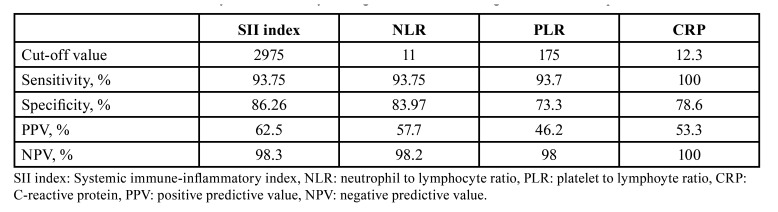
Cut-off value and efficiency of inflammatory serological biomarkers for high-risk of DNI complications.

**Table 4:**
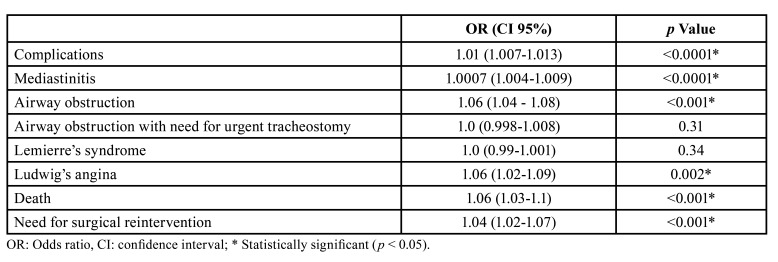
SII index as a high-risk biomarker for DNI complications

## Discussion

DNI represent a significant cause of morbidity and mortality, mainly in underdeveloped countries. DNI of dental origin continue to be common despite the range of available treatment options and worldwide campaigns on the prevention and treatment of dental disease. We performed a retrospective study of 163 patients with DNI diagnosis in a tertiary referral hospital. We compared the predictive value of inflammatory serological biomarkers (SII index, NLR, PLR, and CRP) for DNI complications. NLR, PLR, CRP, and SII index showed high accuracy for DNI complications.

Biomarkers are valuable for the evaluation of inflammatory activity in acute infections. Apoptosis of B-cells and T-cells causes lymphocyte depletion, which is associated with nosocomial infections, bacteremia, and mortality (16,17). Furthermore, neutrophilia combined with systemic neutrophil activation can contribute to organ damage during sepsis (18). As the final event involves an increase in neutrophil counts and a decline in lymphocyte counts, distinct leukocyte ratios have been proposed as markers of infection and possible underlying immune dysfunction (19).

The NLR has been reported as a marker of infection and sepsis, a predictor of appendicitis, cardiovascular diseases, and malignancies (20-24). Previous studies have evaluated the role of NLR in DNI. Baglam *et al*. proposed a cut-off value of 5.4 as a predictor of DNI as a complication of acute bacterial tonsillitis in pediatric patients (25). On the other hand, Gallagher *et al*. proposed the NLR as a biomarker for an increased length of stay in patients with odontogenic DNI (8). Ban *et al*. proposed NLR as an indicator of surgical drainage of DNI. They reported a NLR cut-off value of >8.02 as a predictor for the need for incision and drainage management of DNI. Other associated variables included peripheral rim enhancement of hypodense area on computed tomography, increased CRP (>41.25 mg/L), and increased ESR (>56.6 mm/h) (9). We identify a cut-off value of 11 associated with a high risk of DNI complications.

PLR has also been studied as a marker for distinct cardiovascular (26), sepsis (27), rheumatological (28), and oncological diseases (29). However, its role in DNI was not previously studied. We observed significant higher values in PLR in patients with DNI complications compared with patients with no complications. We report a cut-off value of PLR of 175 with a positive predictive value of 46.2% and a negative predictive value of 98%.

Recently, the SII index was developed as an indicator to reflect the balance of the inflammatory response and immune status of the host. Although the utility of SII index was first described by Hu *et al*. to predict the recurrence of hepatocellular carcinoma (15), its value has been studied in distinct pathologies, such as sepsis (19) and dental infections (30), among others. However, the role of SII index in DNI had not been previously evaluated. In our study, the SII index was associated with a higher risk of mediastinitis, airway obstruction with the need for tracheostomy, need for surgical reintervention, and death. Additionally, patients with complications had a significant higher value of leucocytes, neutrophils, SII index, NLR, PLR, CRP, and ESR. We propose a cut-off SII index value of 2975 as a predictor for high risk of complication in DNI.

As SII index and the studied leukocyte ratios are derived from the values of neutrophils, lymphocytes, and platelets, it can be calculated at admission to identify patients with a high risk of complications. The availability and low cost of these tests make them useful in the evaluation of DNI in the emergency department to predict and evaluate the severity of the disease, possible complications, and the need for early surgical debridement.

Limitations of the study should be considered. This study had a retrospective design and was conducted in a single center. To our knowledge, this is the largest study assessing different inflammatory serological biomarkers and their association with DNI complications.

## Conclusions

Our analysis concluded that the SII index, NLR, and PLR are valuable biomarkers to assess the risk of complications from DNI. SII index showed a high accuracy for prediction of DNI complications with a cut-off value of 2975. The availability and low cost of these tests make them useful in the evaluation of DNI to predict and evaluate the severity of the disease, possible complications, and the need for early surgical debridement.

## References

[1] Russell MD, Russell MS (2018). Urgent Infections of the Head and Neck. Med Clin North Am.

[2] Lee JK, Kim HD, Lim SC (2007). Predisposing factors of complicated deep neck infection: an analysis of 158 cases. Yonsei Med J.

[3] Doležalová H, Zemek J, Tuček L (2015). Deep Neck infections of Odontogenic Origin and Their Clinical Significance. A Retrospective Study from Hradec Králové, Czech Republic. Acta Medica (Hradec Kralove).

[4] Treviño-Gonzalez JL, Maldonado-Chapa F, González-Larios A, Morales-Del Angel JA, Soto-Galindo GA, et al (2022). Deep Neck Infections: Demographic and Clinical Factors Associated with Poor Outcomes. ORL J Otorhinolaryngol Relat Spec.

[5] Adoviča A, Veidere L, Ronis M, Sumeraga G (2017). Deep neck infections: review of 263 cases. Otolaryngol Pol.

[6] Alotaibi N, Cloutier L, Khaldoun E, Bois E, Chirat M, Salvan D (2015). Criteria for admission of odontogenic infections at high risk of deep neck space infection. Eur Ann Otorhinolaryngol Head Neck Dis.

[7] Velhonoja J, Lääveri M, Soukka T, Irjala H, Kinnunen I (2020). Deep neck space infections: an upward trend and changing characteristics. Eur Arch Otorhinolaryngol.

[8] Gallagher N, Collyer J, Bowe CM (2021). Neutrophil to lymphocyte ratio as a prognostic marker of deep neck space infections secondary to odontogenic infection. Br J Oral Maxillofac Surg.

[9] Ban MJ, Jung JY, Kim JW, Park KN, Lee SW, Koh YW (2018). A clinical prediction score to determine surgical drainage of deep neck infection: A retrospective case-control study. Int J Surg.

[10] Şentürk M, Azgın İ, Övet G, Alataş N, Ağırgöl B, Yılmaz E (2016). The role of the mean platelet volume and neutrophil-to-lymphocyte ratio in peritonsillar abscesses. Braz J Otorhinolaryngol.

[11] Shen Y, Huang X, Zhang W (2019). Platelet-to-lymphocyte ratio as a prognostic predictor of mortality for sepsis: interaction effect with disease severity-a retrospective study. BMJ Open.

[12] Kusumoto J, Iwata E, Huang W, Takata N, Tachibana A, Akashi M (2022). Hematologic and inflammatory parameters for determining severity of odontogenic infections at admission: a retrospective study. BMC Infect Dis.

[13] Forget P, Khalifa C, Defour JP, Latinne D, Van Pel MC, De Kock M (2017). What is the normal value of the neutrophil-to-lymphocyte ratio?. BMC Res Notes.

[14] Jaszczura M, Góra A, Grzywna-Rozenek E, Barć-Czarnecka M, Machura E (2019). Analysis of neutrophil to lymphocyte ratio, platelet to lymphocyte ratio and mean platelet volume to platelet count ratio in children with acute stage of immunoglobulin A vasculitis and assessment of their suitability for predicting the course of the disease. Rheumatol Int.

[15] Hu B, Yang XR, Xu Y, Sun YF, Sun C, Guo W (2014). Systemic immune-inflammation index predicts prognosis of patients after curative resection for hepatocellular carcinoma. Clin Cancer Res.

[16] Hotchkiss RS, Tinsley KW, Swanson PE, Schmieg RE Jr, Hui JJ, Chang KC (2001). Sepsis-induced apoptosis causes progressive profound depletion of B and CD4+ T lymphocytes in humans. J Immunol.

[17] Drewry AM, Samra N, Skrupky LP, Fuller BM, Compton SM, Hotchkiss RS (2014). Persistent lymphopenia after diagnosis of sepsis predicts mortality. Shock.

[18] Brown KA, Brain SD, Pearson JD, Edgeworth JD, Lewis SM, Treacher DF (2006). Neutrophils in development of multiple organ failure in sepsis. Lancet.

[19] Russell CD, Parajuli A, Gale HJ, Bulteel NS, Schuetz P, de Jager CPC (2019). The utility of peripheral blood leucocyte ratios as biomarkers in infectious diseases: A systematic review and meta-analysis. J Infect.

[20] Zahorec R (2001). Ratio of neutrophil to lymphocyte counts--rapid and simple parameter of systemic inflammation and stress in critically ill. Bratisl Lek Listy.

[21] Goodman DA, Goodman CB, Monk JS (1995). Use of the neutrophil:lymphocyte ratio in the diagnosis of appendicitis. Am Surg.

[22] Walsh SR, Cook EJ, Goulder F, Justin TA, Keeling N (2005). : Neutrophil-lymphocyte ratio as a prognostic factor in colorectal cancer. J Surg Oncol.

[23] Ommen SR, Hodge DO, Rodeheffer RJ, McGregor CG, Thomson SP, Gibbons RJ (1998). Predictive power of the relative lymphocyte concentration in patients with advanced heart failure. Circulation.

[24] Gibson PH, Croal BL, Cuthbertson BH, Small GR, Ifezulike AI, Gibson G (2007). Preoperative neutrophil-lymphocyte ratio and outcome from coronary artery bypass grafting. Am Heart J.

[25] Baglam T, Binnetoglu A, Yumusakhuylu AC, Gerin F, Demir B, Sari M (2015). Predictive value of the neutrophil-to-lymphocyte ratio in patients with deep neck space infection secondary to acute bacterial tonsillitis. Int J Pediatr Otorhinolaryngol.

[26] Han M, Sun Y, Li N (2022). Relationship between platelet-to-lymphocyte ratio and Coronary Artery Lesion in non-diabetic patients with coronary heart disease. J Pak Med Assoc.

[27] Wang G, Mivefroshan A, Yaghoobpoor S, Khanzadeh S, Siri G, Rahmani F (2022). Prognostic Value of Platelet to Lymphocyte Ratio in Sepsis: A Systematic Review and Meta-analysis. Biomed Res Int.

[28] Erre GL, Paliogiannis P, Castagna F, Mangoni AA, Carru C, Passiu G (2019). Meta-analysis of neutrophil-to-lymphocyte and platelet-to-lymphocyte ratio in rheumatoid arthritis. Eur J Clin Invest.

[29] Li B, Zhou P, Liu Y, Wei H, Yang X, Chen T (2018). Platelet-to-lymphocyte ratio in advanced Cancer: Review and meta-analysis. Clin Chim Acta.

[30] Dogruel F, Gonen ZB, Gunay-Canpolat D, Zararsiz G, Alkan A (2017). The Neutrophil-to-Lymphocyte ratio as a marker of recovery status in patients with severe dental infection. Med Oral Patol Oral Cir Bucal.

